# Mutation at Intronic Repeats of the Ataxia-Telangiectasia Mutated (ATM) Gene and ATM Protein Loss in Primary Gastric Cancer with Microsatellite Instability

**DOI:** 10.1371/journal.pone.0082769

**Published:** 2013-12-06

**Authors:** Hee Sung Kim, Seung Im Choi, Hae Lim Min, Min A. Kim, Woo Ho Kim

**Affiliations:** 1 Department of Pathology, Chung-Ang University College of Medicine, Seoul, Korea; 2 Cancer Research Institute, Seoul National University, Seoul, Korea; 3 Department of Pathology, Seoul National University College of Medicine, Seoul, Korea; Yonsei University College of Medicine, Republic of Korea

## Abstract

Ataxia-telangiectasia mutated (ATM) is a Ser/Thr protein kinase that plays a critical role in DNA damage-induced signaling and initiation of cell cycle checkpoint signaling in response to DNA-damaging agents such as ionizing radiation. We have previously reported the ATM protein loss by immunohistochemistry (IHC) in 16% of human gastric cancer (GC) tissue. We hypothesized that *ATM* gene intron mutations targeted by microsatellite instability (MSI) cause ATM protein loss in a subset of GC. We studied mononucleotide mutations at the intron of *ATM* gene, ATM IHC and MSI in GC. Ten human gastric cancer cell lines were studied for the *ATM* gene mutation at introns, RT-PCR, direct sequencing, and immunohistochemistry. GC tissues of 839 patients were analyzed for MSI and ATM IHC. Among them, 604 cases were analyzed for the *ATM* mutations at introns preceding exon 6, exon 10 and exon 20. Two human GC cell lines (SNU-1 and -638) showed *ATM* intron mutations, deletion in RT-PCR and direct sequencing, and ATM protein loss by IHC. The frequencies of *ATM* mutation, MSI, and ATM protein loss were 12.9% (78/604), 9.2% (81/882) and 15.2% (134/839), respectively. Analysis of associations among MSI, ATM gene mutation, and ATM protein loss revealed highly co-existing *ATM* gene alterations and MSI. *ATM* intron mutation and ATM protein loss were detected in 69.3% (52/75) and 53.3% (40/75) of MSI positive GC. MSI positivity and ATM protein loss were present in 68.4% (52/76) and 48.7% (37/76) of GC with ATM intron mutation. *ATM* mutation and ATM protein loss had characteristics of old age, distal location of tumor, large tumor size, and histologic intestinal type. Our study might be interpreted as that *ATM* gene mutation at intron might be targeted by MSI and lead to ATM protein loss in a selected group of GC.

## Introduction

Ataxia-telangiectasia mutated (ATM) gene is a component of DNA-damage response (DDR) and is activated by DNA double strand breaks (DSBs), and signals the cell-cycle checkpoint to slow the passage of cells through the cycle to facilitate DNA repair. The gene is localized to chromosome 11q22-23. The *ATM* gene is very large, spanning a genomic region of 150 kb, comprising of 66 exons with a coding sequence of 91668 bp. 

ATM is implicated in responses to DSBs that occur physiologically in specialized cell types such as germ cells and lymphocytes. The ATM protein assists cells in recognizing damaged or broken DNA strands. The ATM protein coordinates DNA repair by activating enzymes that fix the broken strands. Efficient repair of damaged DNA strands helps maintain the stability of the cell's genetic information [1]. 

ATM loss was observed in 16% of human GC tissue in a large cohort study [2]. Inactivation of the *ATM* gene may be a frequent event in the development of human gastric cancer (GC); however, the events related to the loss of *ATM* expression in GC could not be explained by the low prevalence of *ATM* mutation. Most of the *ATM* mutations identified in primary GC cell lines and GC tissues were point mutations [3]. 

The DNA mismatch repair (MMR) system corrects the errors that might occur during DNA replication, whereas homologous recombination and non-homologous end joining are involved in the repair of DNA DSB. Mutations in DNA DSB repair genes such as *ATM, MRE11, RAD50, NBS1* and *ATR*, are postulated to play a role in the development of gastrointestinal malignancies with an impaired MMR function. In colorectal tumors with an impaired MMR system, mutations of intronic mononucleotide repeats in *ATM* and *MRE11* were found to result in aberrant splicing, followed by reduced expression of the wild-type protein [4]. 

Approximately 10% of GC is associated with microsatellite instability (MSI). MSI refers to alterations in the number of nucleotide repeats in microsatellite regions located within the coding regions of genes, and results in frameshift mutations. MSI has been identified in the protein coding regions of known target genes including *TGF-βRII, IGFIIR, BAX* [5–7], and *hRAD50* genes [8]. The *hRAD50, BLM*, and *BRACA1* genes have poly (A) mononucleotide repeats within the coding regions, the *hMSH6* has (C)8 tract, the *NBS1* has the (A)7 tract, and the *ATM* has the (T)7 tract. Frameshift mutations of these genes with variable incidences were reported previously [9–11]. 

In addition to the multitude of mutations accumulated in neutral microsatellite sequences, the MSI also generates mutations in cancer genes, i.e., in those genes that play an active role in the multistep process of carcinogenesis. Although repeated sequences in the coding region of genes are shorter than the typical microsatellite loci used for gene mapping, their propensity for undergoing spontaneous slippage errors during replication is still higher than that of nonrepetitive sequences. Mutations of an intronic repeat were reported to induce impaired expression of *MRE11* gene [12,13]. Polypyrimidine repeat mutations in the *ATM* gene may disrupt the correct mRNA splicing [14]. However, there is no evidence that insertion/deletion occurring at these non-coding mononucleotide sequences affect normal ATM protein function in MSI-related GC. In human GC tissue with MSI, the frequency of *ATM* mutation and the association with the loss of protein function is not understood clearly. We have hypothesized that mononucleotide tract alterations in intron of *ATM* gene is associated with loss of ATM protein function in GC with MSI. 

We investigated mutations at the intronic (T)15 within the *ATM* gene intervening sequence (IVS) preceding exon 6, which lead to 22 bp deletion of exon 6 in human gastric cancer cell lines. In the present study, mutations of the *ATM* gene together with the presence of MSI were examined in human GC cell lines and primary GC tissues. Our results showed that *ATM* gene intron mutation was frequent in GC with MSI and was significantly associated with loss of ATM protein function. 

## Materials and Methods

### Ethics Statement

This retrospective study was performed using the samples over the shelves after the pathologic diagnosis, and all of the samples were anonymized before the study. This retrospective study was approved by the Institutional Review Board of Seoul National University Hospital (H-1010-065-336) with waiver of informed consent under the condition of the anonymization. 

### Cell lines and tissue samples

Ten human gastric cancer cell lines- SNU-1, SNU-5, SNU-16, SNU-216, SNU-484, SNU-601, SNU-620, SNU-638, SNU-668 and SNU-719 were obtained from the Korean Cell Line Bank (Seoul, Korea). All cell lines were grown in RPMI1640 supplemented with 10% fetal bovine serum (FBS; Hyclone, Lorgan, UT, USA) and antibiotics (100 U/mL penicillin G and 100 ug/mL streptomycin) at 37°C in a humidified 5% CO_2_ incubator. A cell-line tissue array slide made of harvested and fixed human GC cell lines was obtained for immunohistochemistry (Superbiochips, Korea).

Formalin-fixed, paraffin-embedded GC tissue specimens from 839 patients who underwent curative gastrectomy for primary gastric cancer treatment between 1^st^ January, 2004 and 31^st^ December, 2005 at Seoul National University Hospital were collected. The patients included 577 men and 262 women with a mean age of 57.6 years (range: 4 to 87 years). Information on tumor location was obtained from 835 cases: upper third in 109, middle third in 323, lower third in 383, and the entire stomach in 20 cases. Based on the Lauren classification, the cancers were histologically classified as intestinal type in 381, diffuse type in 327, mixed type in 123, and undetermined in 8 cases. Out of the 839 cases, 165 were early GC and 674 were advanced GC. Lymph node metastasis was present in 531 cases and absent in 308 cases. Two hundred and forty five patients were diagnosed as stage I, 254 as stage II, 291 as stage III, and 85 as stage IV. Adjuvant chemotherapy following surgery was performed on 533 patients, which includes 32 patients in Stage I, 153 in Stage II, 260 in Stage III, and 82 in Stage IV. The mean follow-up period was 49.3 months (range: 0-85 months). Local recurrence was noted in 185 (21.0%), distant metastasis in 85 (9.6%), and death by any cause in 289 out of 882 patients (32.8%). Patient survival data, including dates and causes of death, were obtained from the Korean Central Cancer Registry, Ministry of Health and Welfare, Korea. Standard histopathological examination included assessment of cancer type and pathological tumor stage, according to the criteria established by the 7th Edition of the AJCC Staging Manual [15].

### DNA extraction and MSI determination

Tumor tissue samples on glass slides were examined under a light microscope. An area of the tumor in which the percentage of tumor cells exceeded a minimum of 50% of all cells in that area and a paired area of normal mucosal tissue containing no tumor cells were marked and scraped with a knife blade. The scraped tissue was collected in 1.5 mL microtubes containing 50 μL of tissue lysis buffer (0.5% Tween 20 [Sigma, St Louis, MO], 100 mM Tris HCl buffer [pH 7.6], 1 mM EDTA, and 20 μg proteinase K [Sigma]). The tubes were incubated for 24 to 48 hours at 55°C until the tissue-containing lysis buffer became clear. Proteinase K was inactivated by incubation at 95°C for 10 minutes, and the extracted DNA was stored at −20°C until use. MSI was assessed at the loci BAT25, BAT26, D2S123, D5S346, and D17S250. Tumors were classified as MSI+ when at least 2 (40%) of the 5 microsatellite markers were associated with alleles of altered size in tumor DNA compared with DNA from non-tumor tissue. 

### Detection of mutations of *ATM* at intron mononucleotide repeats

PCR amplification was performed in 10 μL volume containing 1 μL genomic DNA, 5 pmol each of forward and reverse primers, and 5 μL Premix. Thirty-three cycles of PCR were performed with cycle parameter of 95°C for 30 sec, 30 sec at an annealing temperature appropriate for each primer pair and 65°C for 1 min, followed by 10 min extension at 72°C.

Variation of mononucleotide repeat in the *ATM* gene was detected using a PCR-based assay. Genomic DNA was amplified with primers- F-CCTTTTTCTGTATGGGATTATGG, R-TGAACATCTTGTGAAGGTTTCAG for exon6 (T)15 (155 bp), F-TCCTTTTAGTTTGTTAATGTGATGGA, R-GCTGTTGGGGTAGAAGCTGA for exon10 (T)15 (165 bp), and F-GCCAATGGAAGATGTTCTTGA, R-CATCTTGGTCACGACGATACA for exon20 (T)15 (178 bp).

### Detection of aberrant splicing in *ATM*

Total RNA (2.5 μg) from human gastric cancer cell lines prepared by Trizol reagent (GIBCO-BRL) was used for first-strand cDNA synthesis with Superscript II Reverse Transcriptase (GIBCO-BRL) and oligo-d(T)_12-18_ primer. An aliquot of the reaction mixture was amplified by PCR to synthesize various segments of ATM cDNA.

PCR amplification was performed in 20 μL volume containing 1 μL reverse-transcribed cDNA, 5 pmol each of forward and reverse primers, and 10 μL Premix. Thirty-five cycles of PCR were performed with cycle parameter of 95°C for 30 sec, 30 sec at an annealing temperature appropriate for each primer pair and 72°C for 1 min, followed by 10 min extension at 72°C. PCR products were electrophoresed in 2% agarose gel containing ethidium bromide and visualized under UV light. β-actin was used as an internal standard.

Three RT-PCR primer pairs derived from *ATM* sequences (GenBank NM_000051) used were F-TGGTGCTATTTACGGAGCTG and R-TTCGAAAGTTGACAGCCAAA to detect transcripts of exon 5-7 (product size: 347 bp), F-TCCCTTGCAAAAGGAAGAAA and R-TACTGGTGGTCAGTGCCAAA for exon 9-11 (product size: 504 bp), and F-TGGAGAAGAGTACCCCTTGC and R-GATTGACTCTGCAGCCAACA for exon 19-22 (product size: 415 bp). 

### Sequencing analysis

Sequencing was carried out by the dideoxy-chain termination method using DNA sequencing kit (Applied Biosystems) and the ABI PRISM 310 Genetic Analyzer. Amplified PCR products were purified enzymatically using a presequencing kit (Amersham Life Science, Cleveland, OH, USA), according to the manufacturer’s instructions, and then directly sequenced using BigDye terminator method (Applied Biosystems, Foster City, CA, USA). Sequencing reactions were run on an ABI 3100 automated sequencer (Applied Biosystems, Biosystems, Foster City, CA, USA) and the data obtained were analyzed using DNA sequencing analysis 3.7 software (Applied Biosystems, Foster City, CA, USA). The new data has been deposited in GenBank (accession number: KF704396).

### Immunohistochemistry

Representative tumor-block sections and cell-line array sections of 4-μm thickness were deparaffinized and rehydrated in graded alcohol. Antigen retrieval was achieved by pressure -cooking the samples in 0.01 mol/L citrate buffer for 5 min. The rabbit monoclonal antibody (clone Y170 obtained from Epitomics, Burlingame, CA, USA) was used for immunohistochemistry. ATM was stained in the nucleus of normal gastric mucosa, stromal cells, and lymphocytes. Intensity was graded as 0 (entirely negative), ± (equivocal staining, signal observed only at high-power microscopy with ×40 eyepiece), +/3 (weak positive), ++/3 (moderately positive) and +++/3 (strongly positive, almost equal to lymphocytes and neutrophils). The criteria for negative cases were set as less than 10% of cells stained as weak positive (+/3) or higher intensity, that is, more than 90% of cells showing totally negative (0) or equivocal staining (±) [2].

### Statistical analysis

The SPSS 15.0 package was used for statistical analyses. Chi-square test and Cox proportional hazards model were used. All P-values are two sided and P values <0.05 were considered as significant for the statistical methods in this study.

## Results

### Mutation at intronic mononucleotide repeats of the ATM gene in human gastric cancer cell lines

**Figure 1:**
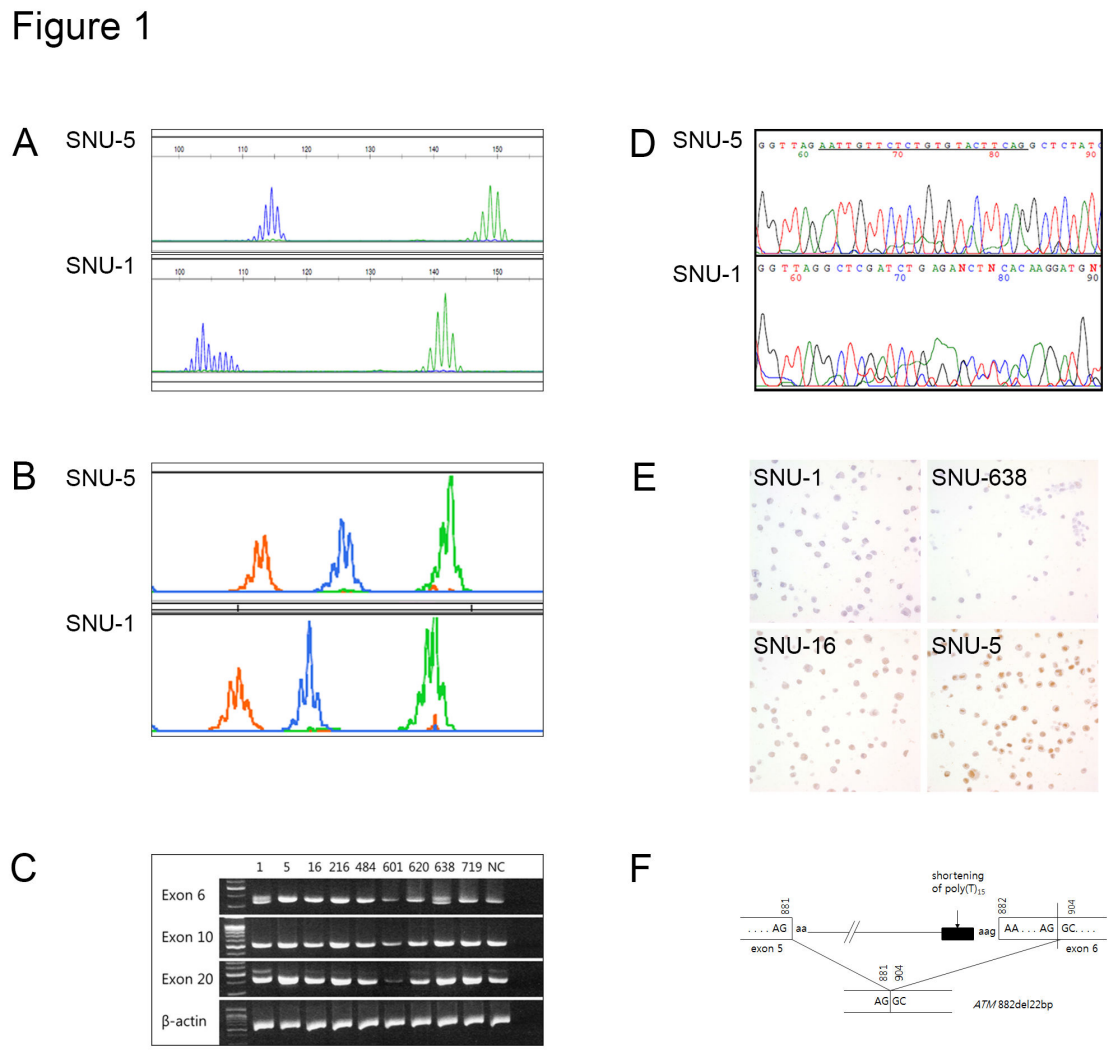
Microsatellite instability (MSI) and *ATM* gene mutations at introns in human gastric cancer cell lines. (A) BAT26 (blue) and BAT25 (green) showed stable pattern in SNU-5 (upper) and unstable pattern in SNU-1 (lower). (B) ATM gene PCR products inintervening sequence (IVS6) (orange), IVS10 (blue), IVS20 (green) showed normal length in SNU-5 (upper) and shortening in SNU-1 (lower). (C) RT-PCR of *ATM* exon 6, exon 10, and exon 20 in gastric cancer cell lines. SNU-1 and 638 showed 22-bp deletion at exon 6. (D) Direct sequencing of IVS 6. SNU-5 has normal sequence and SNU-1 has 22 deletion at underlined sequences. (E) Immunohistochemistry of ATM protein in gastric cancer cell-lines. Negative in SNU-1, equivocal in SNU-638, mild positive in SNU-16 and strong positive in SNU-5. (F) Schematic representation of a 22-bp deletion of exon 6 of *ATM* (882del22).

Among 10 human gastric cancer cell lines, SNU-1 and SNU-638 were characterized as MSI positive, *ATM* gene mutations positive and ATM loss by IHC. In SNU-1 and SNU-638, 22 base pair deletion of exon 6 was detected by RT-PCR and it was confirmed in subsequent sequence analysis (Figure 1). SNU-5, SNU-16, SNU-484, SNU-601, SNU-620, SNU-668, and SNU-719 were MSI negative and had wild type *ATM* gene. Although SNU-216 was MSI negative, it harbored mutations in the *ATM* gene, and had strong ATM expression detected by IHC (Table 1). 

**Table 1:** Microsatellite instability status and *ATM* gene profiles of human gastric cancer cell lines.

	MSI		*ATM* gene mutation of intronic mononucleotide repeats			*ATM* sequencing			*ATM* RT-PCR			ATM IHC
	Bat 26	Bat 25	IVS 6	IVS 10	IVS 20	Exon 6	Exon 10	Exon 20	Exon 6	Exon 10	Exon 20	
SNU-1	+	+	+	+	+	22-bp del	no	no	22-bp del	no	no	Negative
SNU-5	-	-	-	-	-	no	no	no	no	no	no	Positive
SNU-16	-	-	-	-	-	nd	nd	nd	no	no	no	Positive
SNU-216	-	-	+	+	+	no	no	no	no	no	no	Positive
SNU-484	-	-	-	-	-	nd	nd	nd	no	no	no	Positive
SNU-601	-	-	-	-	-	nd	nd	nd	no	no	no	Positive
SNU-620	-	-	-	-	-	nd	nd	nd	no	no	no	Positive
SNU-638	+	+	+	+	+	22-bp del	no	no	22-bp del	no	no	Negative
SNU-668	-	-	-	-	-	nd	nd	nd	no	no	no	Positive
SNU-719	-	-	-	-	-	nd	nd	nd	no	no	no	Positive

IVS, intervening sequence; IHC, immunohistochemistry.

### Mutation at intron mononucleotide repeats of the *ATM* gene and microsatellite instability in gastric cancer tissue

**Figure 2:**
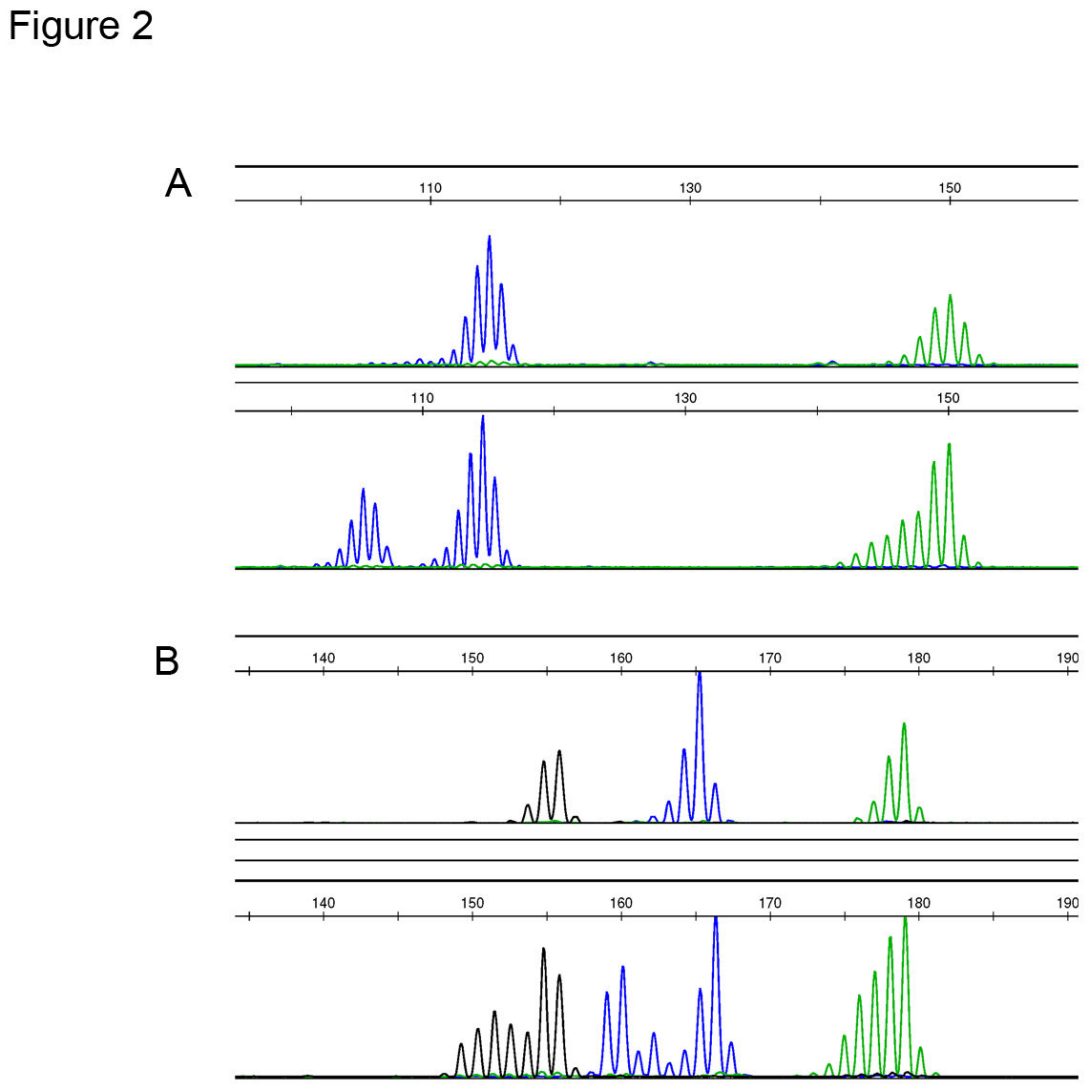
Microsatellite instability and *ATM* gene mutations at introns preceding exon 6, exon 10 and exon 20 in human gastric cancer tissue (A) BAT26 and BAT25. Lower lane showed instability in BAT26 (blue) and BAT25 (green). (B) Lower lane showed mutation at intervening sequence 6 (IVS6) (blue).

**Figure 3:**
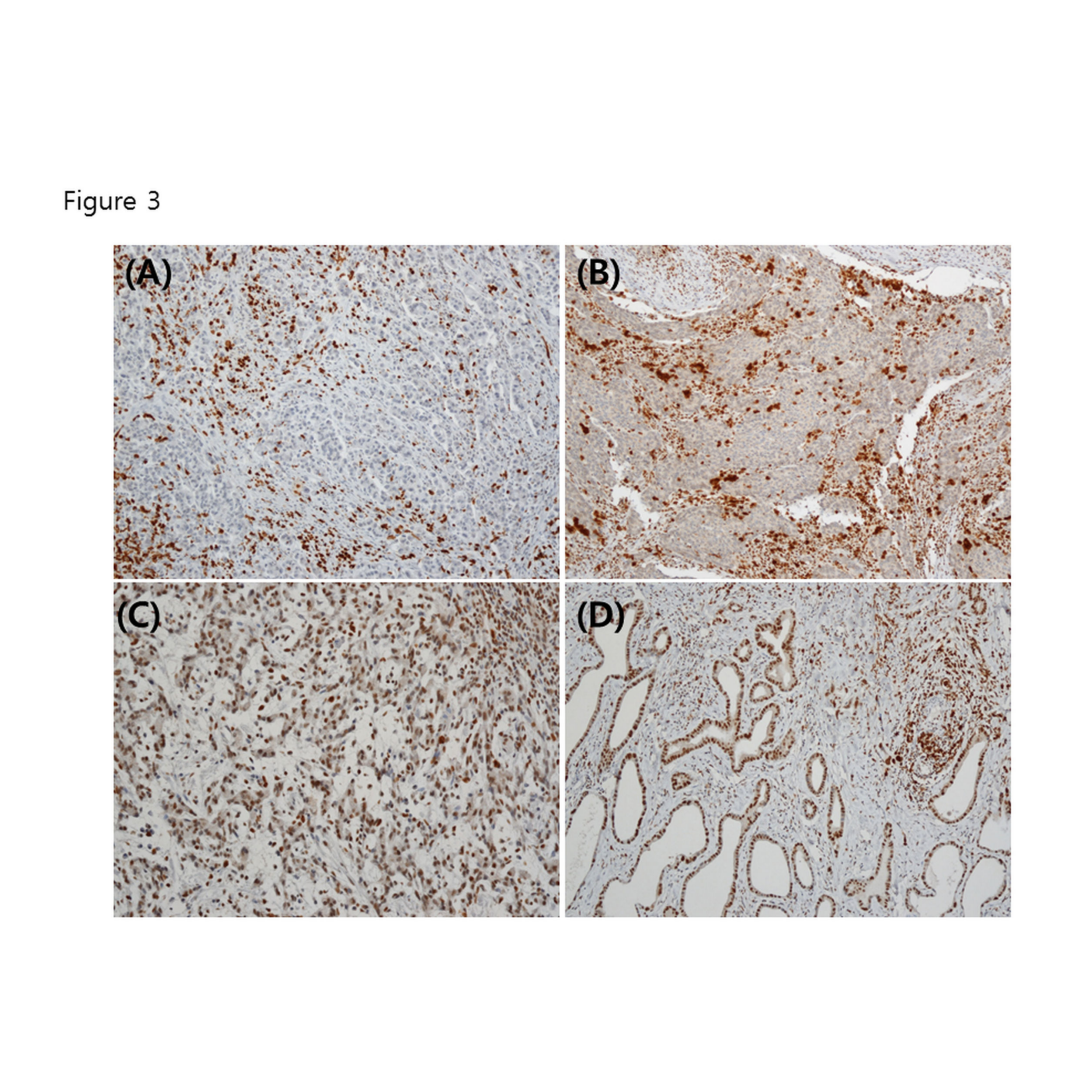
ATM immunohistochemistry in gastric cancer tissue. (A) Negative. (B) Equivocal (+/-). (C) Weak positive (1+~2+). (D) Strong positive (3+).

**Figure 4:**
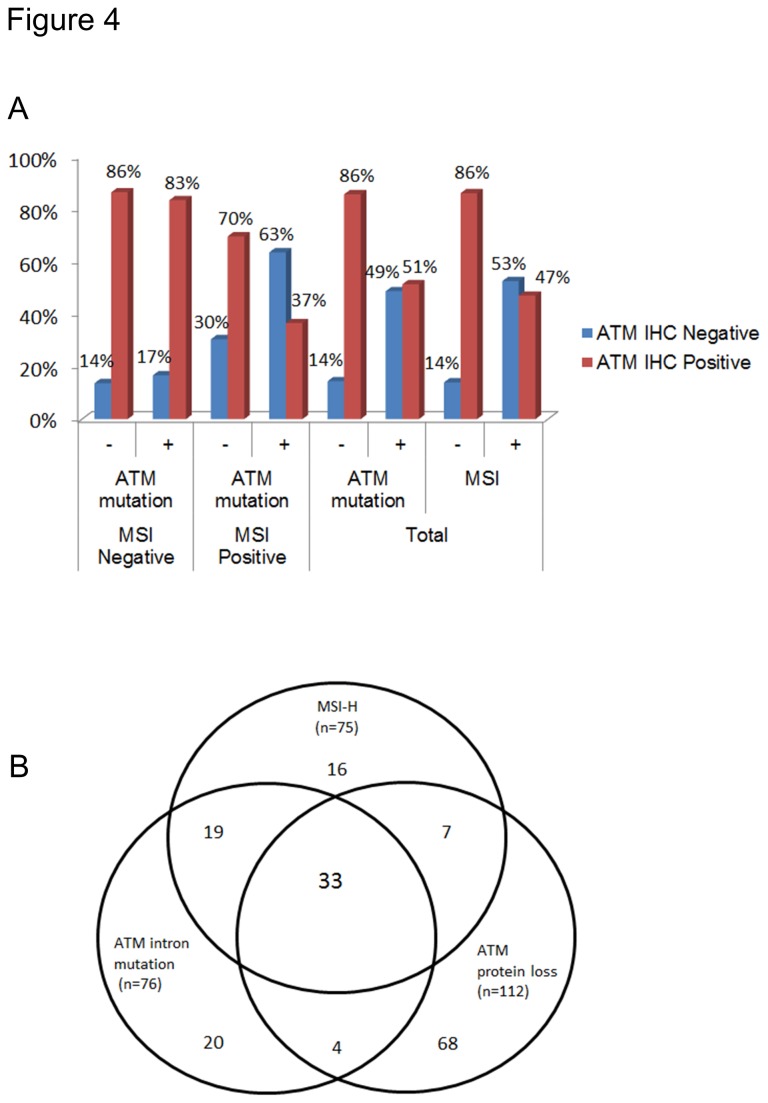
Associations among microsatellite instability (MSI), *ATM* intron mutation and ATM protein loss in the gastric cancers. (A) Frequencies of ATM protein loss by immunohistochemistry in gastric cancer subgroups by MSI status and *ATM* intron mutation. (B) Venn diagram showing the number of gastric cancer cases with overlapping molecular characteristics among high-level microsatellite instability (MSI-H), *ATM* intron mutation, and ATM protein loss.

The frequency of intronic mutation in *ATM* gene was determined as 12.9% (78/604). The frequency of the *ATM* gene mutation was 9.3% (50/540) in intron preceding IVS 6; 11.0% (59/534) in intron preceding IVS 10 and 8.8% (46/523) in intron preceding IVS 20; among them, IVS 6 and IVS 10 overlapped in 39, IVS 10 and IVS 20 in 35, IVS 10 and IVS 20 in 34, IVS 6, IVS 10 and IVS 20 in 31 cases (Table 2). The frequencies of MSI positivity was 9.2% (81/882) and ATM protein loss was 15.2% (134/839) in our study of GC. In 596 cases, the associations among MSI (Figure 2A), ATM gene mutation (Figure 2B), and ATM protein loss (Figure 3) were analyzed. *ATM* intron mutation and ATM protein loss were detected in 69.3% (52/75) and 53.3% (40/75) of MSI positive GC. MSI positivity and ATM protein loss were present in 68.4% (52/76) and 48.7% (37/76) of GC with ATM intron mutation. MSI positivity and ATM intron mutation were detected in 35.7% (40/112) and 33.0% (37/112) of GC with ATM protein loss (Table 3). The frequency of ATM IHC results showed a significantly higher ATM loss in a subgroup of MSI positive and *ATM* mutation positive GC (Figure 4A). Thirty three of 596 cases (5.5%) have the all three molecular features (Figure 4B).

**Table 2:** Incidencies of *ATM* gene intron mutation in the gastric cancers (GC).

Location	Repeats (wild type)	Intron mutation in GC	Intron mutation in MSI positive GC	Intron mutation in ATM IHC negative GC
*ATM* IVS 6	(T)_15_	50/540 (9.3%)	42/50 (84.0%)	29/111 (26.1%)
*ATM* IVS 10	(T)_15_	59/534 (11.0%)	47/59 (78.7%)	29/111 (26.1%)
*ATM* IVS 20	(T)_15_	46/523 (8.8%)	34/46 (73.9%)	22/107 (20.6%)
*ATM* IVS 6 or IVS 10 or IVS 20	(T)_15_	78/604 (12.9%)	53/76 (69.7%)	37/112 (33.0%)

IVS, intervening sequence; MSI, microsatellite instability; IHC, immunohistochemistry.

**Table 3:** Frequency of ATM protein expression in subgroups according to microsatellite instability (MSI) status and *ATM* intron mutation.

			ATM IHC	
MSI	*ATM* mutation	N	Negative	Positive
Negative	Negative	497	68 (13.7%)	429 (86.3%)
	Positive	24	4 (16.7%)	20 (83.3%)
	Subtotal	521	72 (13.8%)	449 (86.2%)
Positive	Negative	23	7 (30.4%)	16 (69.6%)
	Positive	52	33 (63.5%)	19 (36.5%)
	Subtotal	75	40 (53.3%)	35 (46.7%)
	Negative	520	75 (14.4%)	445 (85.6%)
	Positive	76	37 (48.7%)	39 (51.3%)
	Total	596	112 (18.8%)	484 (81.2%)

### Clinicopathologic correlations of *ATM* gene mutation in GC

GC patients with *ATM* gene mutation were associated with old age, large tumor size, distal location, intestinal type, deeper invasion, and lower recurrence rate. GC patients with negative ATM IHC were associated with old age, large tumor size, well to moderately differentiated type, and intestinal type of Lauren’s classification (Table S1). Chi-square test *ATM* gene mutation status in ATM IHC negative and positive groups with clinicopathologic variables was performed. In the ATM IHC negative group, *ATM* mutation positive GC were associated with old age (P=0.032) and distal location (P=0.045). In the ATM IHC positive group, *ATM* gene mutation was associated with well or moderately differentiated type of GC (P = 0.022) (Table S1).

### Survival analysis

The Cox proportional hazards model was used in both univariate and multivariate tests. Univariate Cox regression analysis for overall survival and disease free survival showed significant predictive value of some established prognostic factors such as lymph node metastasis, depth of tumor, and Lauren’s classification of histologic type. The patient group who received adjuvant chemotherpy had a significantly high relative risk for overall (OS) and disease free survival (DFS) (P < 0.001, Relative risk = 3.82 [95.0% CI, 2.81-5.18] for OS and P < 0.001, Relative risk = 5.63 [95.0% CI, 3.69-8.58] for DFS). However, no significant trends of relative risk (RR) was observed for *ATM* intron mutation, MSI, or ATM IHC loss. Multivariate analysis using variables of depth of tumor, lymph node status, Lauren’s classification of histologic type, adjuvant chemotherapy, *ATM* intron mutation, and ATM protein expression was performed. ATM intron mutation has a lower relative risk with a borderline significance for overall survival (P = 0.05, Relative risk = 0.60, [95.0% CI 0.36-1.00]) (Table 4).

**Table 4:** Multivariate Cox regression analysis.

	Overall Survival					Disease Free Survival			
	Relative risk	95.0% CI	P value	N		Relative risk	95.0% CI	P value	N
Advanced Gastric Cancer	8.26	3.00-22.75	0.00	596		5.05	1.81-14.13	0.00	544
Lymph node positive	6.66	3.47-12.77	0.00	596		3.20	1.58-6.48	0.00	544
Diffuse type histology	1.43	1.06-1.94	0.02	596		1.61	1.09-2.36	0.02	544
Adjuvant chemotherapy	0.99	0.57-1.73	0.98	596		2.17	1.00-4.72	0.05	544
*ATM* intron mutation	0.60	0.36-1.00	0.05	596		0.56	0.29-1.08	0.08	544
ATM protein loss	1.19	0.82-1.73	0.35	596		1.10	0.67-1.81	0.70	544

## Discussion

Our results showed that mutations of an intron repeat in the *ATM* gene result in aberrant splicing, leading to 22 bp deletion and ATM protein expression loss in GC cell lines. These results are consistent with the previous report [13] regarding the identification of a splicing defect in the *MRE11* precursor transcript linked to mutations by a poly(T)11 repeat within intervening sequence 4 (IVS-4), exclusively in MMR-deficient cancer cell lines and primary tumors. Our results revealed that ATM protein loss in GC significantly correlate with the presence of intronic gene mutation in *ATM*. 

In Ataxia-Telangiectasia patients, 70%–90% of *ATM* mutations are nonsense, frame shift, or splicing mutations that truncate the protein [16]. According to COSMIC databases (http://www.sanger.ac.uk/cosmic), mutations at coding regions of *ATM* gene were detected in 5% (433 of 9249) of various cancers. Among them stomach cancer showed mutation only in 1.35% (1 of 74). Although *ATM* gene mutation in GC was reported previously [3,9], comprehensive explanation was limited by the low frequency of mutation and relatively high incidence of functional loss of ATM [2]. In our study, about 70% of MSI positive GC showed significant *ATM* gene intronic mutation associated with ATM protein loss. Our results strongly support that intronic mutation of the *ATM* with underlying MSI is one of the major mechanisms of ATM loss in human GC. ATM loss is attributed to aberrant splicing associated with intronic shortening in MSI positive colon-cancer cell lines. Ejima et al. searched *ATM* gene mutations using 62 pairs of oligonucleotide primers, corresponding to the region ranging from exon 4 through exon 65 of *ATM* and its flanking introns. Shortening of mononucleotide tracts of *ATM* introns accounted for 34 (87%) of 39 intronic changes. Thirty-one of them were found in the 5 colon-tumor cell lines having MSI (LS180, HCT15, HCT116, SW48, and LoVo). They also showed low expression of ATM protein in MSI positive colorectal cancer cell lines [14]. The ATM expression is significantly reduced in many breast carcinomas, and found no *ATM* mutation in that cell line [17]. This indicates different features of *ATM* gene alterations between gastrointestinal and breast cancers.

In addition to mutations, tumor suppressor genes can be inactivated by methylation of cytosine residues within CpG islands which are located in the promoter of many genes. *ATM* gene is a novel target for epigenetic silencing through inappropriate methylation of its proximal promoter region correlates with increased radiosensitivity and low protein expression in a human colorectal tumor cell line [18] and human glioma cell line [18]. without any *ATM* gene mutations.

The MSI targeting of the *ATM* gene represent a link between MSI and DNA repair in GC. Several studies indicate that functional loss of ATM is increased in MSI and human *ATM* gene is a major target for inactivation in MSI positive GC cell lines and tissues [19,20]. The reason for strikingly high incidence of *ATM* gene intron mutation in MSI positive GC might be due to the mononucleotide repeats (T)15, and a length of 13 nucleotides or more were particularly susceptible to this targeted shortening [14]. The frequency of *ATM* gene frameshift mutation (T)7 in exon 6 coding sequence in MSI positive GC was reported to be only 6% (2/36) [8], which is much lower than our observation. 

It is reported that ATM deficiency sensitizes cells to the growth-inhibitory effects of PARP inhibitor, olaparib, in gastric cancer cells with reduced *ATM* expression [21]. Poly (ADP-ribose) polymerase inhibitors are currently evaluated in clinical trials against a variety of cancers, including in colon carcinoma [22]. In GC, ATM deficiency is known to be a predictor of response to PARP inhibitor [23–25]. A marker of homologous recombination, Rad 51, is known to be predictive marker of neoadjuvant anthracylcine-based chemotherapy in breast cancer [26] as well as of PARP inhibitor in GC [24].

Our results showed that ATM protein loss is not an aggressive factor of GC, although it is significantly associated with unfavorable trend of HR in MSI negative GC. This is comparable with other studies that report ATM protein loss as an adverse prognostic factor [20]. In our studies, *ATM* mutation and ATM negative GC had distinct clinicopathologic characteristics; however, this group did not show any survival difference with others. In MSI negative group, HR of GC with ATM protein loss was significantly high, however, in MSI positive group, HR in GC with ATM protein loss was not significant. This indicates that MSI is a potential molecular factor affecting biological behavior of GC. Thus, our study supports that MSI status could influence the sensitivity to PARP inhibitor by affecting the status of DNA damage response protein expression through mononucleotide repeats alterations. 

In summary, *ATM* gene mutation in GC is 12.9%, and 33.0% of GC with *ATM* gene mutation is associated with ATM protein loss. MSI positive GC showed *ATM* mutation in 69.7%. Our results suggest that the outcome of ATM loss and sensitivity to chemotherapeutic compound, olaparib, might be better understood if cases are stratified based on *ATM* gene mutation status or MSI. 

## Conclusions

Our study revealed *ATM* gene intron mutation is present in 69% of MSI positive GC and 49% of *ATM* intron mutation is associated with loss of ATM protein. These suggest the *ATM* gene intron mutation targeted by microsatellite instability is associated with ATM protein loss in a subset of human gastric cancer. 

## Supporting Information

Table S1 **Clinicopathologic correlations of gastric carcinoma with the ATM gene mutation and ATM protein expression.**
(DOCX)
