# Supplementary material for: Mutation at Intronic Repeats of the Ataxia-Telangiectasia Mutated (ATM) Gene and ATM Protein Loss in Primary Gastric Cancer with Microsatellite Instability
Source: PLoS One. 2013 Dec 6;8(12):e82769. doi: 10.1371/journal.pone.0082769 (PMC3855840; doi:10.1371/journal.pone.0082769)
Supplement: Table S1 — Clinicopathologic correlations of gastric carcinoma with the ATM gene mutation and ATM protein expression. (DOCX) [file pone.0082769.s001.docx]

Table S1. Clinicopathologic correlations of gastric carcinoma with the *ATM* gene mutation and ATM protein expression

|  |  |  |  |  |  |  |  |  |  | IHC (-) |  |  |  |  | IHC (+) |  |  |  |  |
| --- | --- | --- | --- | --- | --- | --- | --- | --- | --- | --- | --- | --- | --- | --- | --- | --- | --- | --- | --- |
|  |  | Total | M (+) | | P | Total | IHC (-) |  | P | M (-) |  | M (+) |  | P | M (-) |  | M (+) |  | P |
|  |  | N | N | % |  | N | N | % |  | N | % | N | % |  | N | % | N | % |  |
|  | Total | 604 | 78 | 13 |  | 839 | 134 | 16 |  | 75 | 67 | 37 | 33 |  | 445 | 92 | 39 | 8 |  |
| Sex | Male | 415 | 47 | 11 | 0.085 | 577 | 84 | 15 | 0.097 | 50 | 74 | 18 | 27 | 0.066 | 315 | 92 | 28 | 8 | 0.894 |
|  | Female | 189 | 31 | 16 |  | 262 | 50 | 19 |  | 25 | 57 | 19 | 43 |  | 130 | 92 | 11 | 8 |  |
| Age (yr) | <60 | 304 | 25 | 8 | 0.001 | 427 | 45 | 11 | <0.001 | 34 | 79 | 9 | 21 | 0.032 | 242 | 94 | 16 | 6 | 0.109 |
|  | ≥60 | 300 | 53 | 18 |  | 412 | 89 | 22 |  | 41 | 59 | 28 | 41 |  | 203 | 90 | 23 | 10 |  |
| Tumor size | <4cm | 205 | 16 | 8 | 0.007 | 286 | 31 | 11 | 0.004 | 18 | 78 | 5 | 22 | 0.196 | 167 | 94 | 10 | 6 | 0.139 |
|  | ≥4cm | 399 | 62 | 16 |  | 553 | 103 | 19 |  | 57 | 64 | 32 | 36 |  | 278 | 91 | 29 | 9 |  |
| Location | Upper | 93 | 2 | 2 | 0.009 | 109 | 15 | 14 | 0.009 | 13 | 100 | 0 | 0 | 0.045 | 78 | 98 | 2 | 3 | 0.111 |
|  | Middle | 211 | 32 | 15 |  | 323 | 40 | 12 |  | 20 | 67 | 10 | 33 |  | 155 | 89 | 20 | 11 |  |
|  | Lower | 280 | 44 | 16 |  | 383 | 76 | 20 |  | 39 | 59 | 27 | 41 |  | 195 | 92 | 17 | 8 |  |
|  | Entire | 18 | 0 | 0 |  | 20 | 2 | 10 |  | 2 | 100 | 0 | 0 |  | 16 | 100 | 0 | 0 |  |
| WHO | WD tubular | 45 | 10 | 22 | 0.004 | 52 | 9 | 17 | <0.001 | 4 | 57 | 3 | 43 | 0.256 | 31 | 82 | 7 | 18 | 0.022 |
|  | MD tubular | 202 | 29 | 14 |  | 282 | 54 | 19 |  | 26 | 59 | 18 | 41 |  | 145 | 94 | 10 | 7 |  |
|  | PD tubular | 233 | 32 | 14 |  | 335 | 47 | 14 |  | 29 | 73 | 11 | 28 |  | 169 | 89 | 20 | 11 |  |
|  | Signet ring cell | 91 | 1 | 1 |  | 122 | 9 | 7 |  | 8 | 100 | 0 | 0 |  | 81 | 99 | 1 | 1 |  |
|  | Undifferentiated | 6 | 2 | 33 |  | 6 | 4 | 67 |  | 2 | 50 | 2 | 50 |  | 2 | 100 | 0 | 0 |  |
|  | Other | 27 | 4 | 15 |  | 42 | 11 | 26 |  | 6 | 67 | 3 | 33 |  | 17 | 94 | 1 | 6 |  |
| Lauren | Intestinal | 279 | 49 | 18 | 0.002 | 381 | 75 | 20 | 0.001 | 39 | 63 | 23 | 37 | 0.338 | 188 | 88 | 25 | 12 | 0.067 |
|  | Diffuse | 231 | 19 | 8 |  | 327 | 38 | 12 |  | 23 | 77 | 7 | 23 |  | 186 | 94 | 11 | 6 |  |
|  | Mixed | 89 | 8 | 9 |  | 123 | 17 | 14 |  | 12 | 71 | 5 | 29 |  | 69 | 96 | 3 | 4 |  |
|  | Undetermined | 5 | 2 | 40 |  | 8 | 4 | 50 |  | 1 | 33 | 2 | 67 |  | 2 | 100 | 0 | 0 |  |
| T stage | T1 | 131 | 10 | 8 | 0.047 | 165 | 24 | 15 | 0.638 | 14 | 78 | 4 | 22 | 0.305 | 106 | 95 | 6 | 5 | 0.226 |
|  | T2 | 105 | 21 | 20 |  | 167 | 23 | 14 |  | 11 | 52 | 10 | 48 |  | 72 | 87 | 11 | 13 |  |
|  | T3 | 242 | 31 | 13 |  | 337 | 60 | 18 |  | 37 | 71 | 15 | 29 |  | 171 | 92 | 15 | 8 |  |
|  | T4 | 126 | 16 | 13 |  | 170 | 27 | 16 |  | 13 | 62 | 8 | 38 |  | 96 | 93 | 7 | 7 |  |
| Lymph node | Positive | 231 | 30 | 13 | 0.966 | 308 | 40 | 13 | 0.072 | 25 | 68 | 12 | 32 | 0.924 | 172 | 91 | 17 | 9 | 0.544 |
|  | Negative | 373 | 48 | 13 |  | 531 | 94 | 18 |  | 50 | 67 | 25 | 33 |  | 273 | 93 | 22 | 8 |  |
| AJCC | I | 173 | 21 | 12 | 0.694 | 235 | 33 | 14 | 0.361 | 18 | 69 | 8 | 31 | 0.566 | 132 | 91 | 13 | 9 | 0.749 |
|  | II | 162 | 25 | 15 |  | 238 | 34 | 14 |  | 19 | 61 | 12 | 39 |  | 116 | 91 | 12 | 9 |  |
|  | III | 193 | 25 | 13 |  | 278 | 53 | 19 |  | 28 | 65 | 15 | 35 |  | 138 | 94 | 9 | 6 |  |
|  | IV | 69 | 7 | 10 |  | 81 | 14 | 17 |  | 10 | 83 | 2 | 17 |  | 52 | 91 | 5 | 9 |  |
| Recurrence | No | 424 | 64 | 15 | 0.048 | 594 | 92 | 16 | 0.800 | 49 | 62 | 30 | 38 | 0.078 | 306 | 91 | 32 | 10 | 0.216 |
|  | Recurrence | 123 | 11 | 9 |  | 178 | 31 | 17 |  | 18 | 72 | 7 | 28 |  | 93 | 96 | 4 | 4 |  |
|  | R1 resection | 54 | 3 | 6 |  | 64 | 11 | 17 |  | 8 | 100 | 0 | 0 |  | 43 | 94 | 3 | 7 |  |

M, Mutation; IHC, Immunohistochemistry
